# Circulating MMP11 and specific antibody immune response in breast and prostate cancer patients

**DOI:** 10.1186/1479-5876-12-54

**Published:** 2014-02-24

**Authors:** Giuseppe Roscilli, Manuela Cappelletti, Claudia De Vitis, Gennaro Ciliberto, Arianna Di Napoli, Luigi Ruco, Rita Mancini, Luigi Aurisicchio

**Affiliations:** 1 Takis, Rome, 00128, Italy; 2 Department of Clinical and Experimental Medicine, Laboratory of Surgery “P.Valdoni”, University “La Sapienza”, Rome, Italy; 3 IRCCS National Cancer Institute “G. Pascale”, Naples, Italy; 4 Department of Clinical and Experimental Medicine, University “La Sapienza”, Azienda Ospedaliera S. Andrea, Rome, Italy; 5 Biogem s.c. a r.l, 80123 Ariano Irpino, AV, Italy

**Keywords:** MMP11, Tumor stroma, Immune response

## Abstract

**Background:**

Tumor Associated Antigens are characterized by spontaneous immune response in cancer patients as a consequence of overexpression and epitope-presentation on MHC class I/II machinery. Matrix Metalloprotease 11 (MMP11) expression has been associated with poor prognosis for several cancer types, including breast and prostate cancer.

**Methods:**

MMP11 expression was determined by immunoistochemistry in breast and prostate cancer samples. Circulating MMP11 protein as well as the spontaneous immune responses against MMP11 were analyzed in a set of breast and prostate cancer patients.

**Results:**

In plasma samples MMP11 protein was present in 5/13 breast cancer patients and in 1/12 prostate cancer patients. An antibody response was observed in 7/13 breast cancer patients and in 3/12 prostate cancer patients.

**Conclusions:**

These findings further suggest MMP11 as a promising biomarker for these tumor types and a suitable target for cancer immunotherapy strategies.

## Background

Cancer is essentially considered a complex cell disease. However, in recent decades increasing research of the tumoral microenvironment has revealed the crucial role of stromal cells and host’s immune system in determining the neoplastic phenotype [1-3]. Therefore, cancer could be explained, at least in part, as a complex interaction with different cell types and an abnormal immune system tolerance to uncontrolled cancer cells.

The therapeutic potential of targeting tumor stroma has been shown in several preclinical and clinical studies. T cells and antibodies represent an important alternative approach to the effective control of tumor growth, particularly in the absence of direct targeting of cancer cells [4]. Cellular targets of active immune interventions include cancer-associated fibroblasts, infiltrating macrophages/histiocytes, and tumor endothelial cells. Antigens as carbonic anhydrase IX or fibroblast activation protein (FAP) α suggest that vaccination against stromal antigens is a feasible approach for anticancer therapy [5]. Matrix metalloproteases (MMP) are overexpressed and contribute to neoplastic phenotype and metastatic activity [6,7]. Immunologic targeting of MMPs has been suggested in several studies. The antitumoral effects of a vaccine against MMP2 have been reported [8]. MMP7 was identified as a novel broadly expressed tumor-associated antigen and a T-cell epitope derived from this protein was proposed as candidate for vaccine development [9]. These observations show that MMPs are valid candidates for antigen-specific immunotherapy.

Recently, our group has shown that MMP11 may represent an ideal self-antigen for immunotherapy. It is differentially expressed in tumor versus normal tissue [10], although it is unclear if it is expressed in cancer cells or in the supporting stroma. A genetic vaccine against MMP11 based on DNA electro-gene-transfer technology was able to break immune tolerance and exert antitumor effects in a chemically-induced colon adenocarcinoma mouse model [10]. A strong interferon-γ/cytotoxic cell-mediated and antibody response was elicited by this vaccine. Levels of MMP11 expression may be used to identify patients at greatest risk for cancer recurrence, in breast carcinoma, pancreatic tumors [11] and colon cancer [12]. Furthermore, the prognostic significance of MMP11 expression was further confirmed for breast cancer [13] and shown for prostate cancer [14]. MMP11 is processed intracellularly and secreted as an active form [15]. MMP11 thus differs from other MMPs that are expressed as proenzymes and processed to active forms through proteolytic cleavage activated extracellularly, indicating that MMP11 may have a unique role in tumor development and progression [16].

Tumor Associated Antigens (TAAs) are characterized by spontaneous immune response in cancer patients as a consequence of overexpression, shedding and epitope-presentation on MHC class I/II machinery. For instance, spontaneous antibodies against HER2, Carcinoembryonic Antigen (CEA), p53 and cyclin B1 are commonly detected in patients affected by breast cancer [17,18] and multiple autoantibodies are dectected in Hepatocarcinoma patients [19]. However, these antibodies do not reach a titer sufficient to exert antitumor effects.

To assess whether the overexpression of MMP11 in cancer patients may spontaneously induce a specific immune response, in this study we have confirmed the expression of the protein in breast and prostate tumor microenvironment and then we have measured circulating MMP11 protein and anti-MMP11 antibodies in a set of breast and prostate cancer patients. Our findings validate MMP11 as a potential biomarker for these tumor types and a suitable target for cancer immunotherapy strategies.

## Methods

### Tissue specimens and immunoistochemical staining

Archival pathological tissue specimens were obtained at Sant’Andrea Hospital in Rome from 11 patients with HER2-positive invasive ductal carcinoma of the breast and from 5 invasive prostate adenocarcinomas. Paraffin tissue sections were immunostained with a rabbit monoclonal antibody anti-MMP11 (clone EP1259Y, dilution 1:200, Abcam, Cambridge, UK) using an automated immunostainer (DAKO, Denmark).

### Human plasma samples

They were obtained from the Pathology Unit, Saint Savas Hospital and were kindly provided by Dr CN Baxevanis. All tissue samples were clinicopathologically assessed. Samples of healthy donors were used as negative controls and obtained at Sant’Andrea Hospital, University of Rome. All patients voluntarily provided their blood for research purposes.

### Detection of MMP11 protein

MMP11 protein in plasma samples was detected by ELISA. MMP11 ELISA assay was first optimized using extracts from HeLa cells (ATCC) transfected in 6 cm dishes with 5 μg pV1J-hMMP11 plasmid [10] using Lipofectamine 2000 (Gibco). 48 hr later, cells were lysed in RIPA buffer containing Protease inhibitors (cOmplete Protease Inhibitor Cocktail, Roche). Briefly cell pellet (about 1×10^6^ cells) was resuspended in 200 μL of cold RIPA buffer and incubated on ice for 15 min. Lysate was then cleared by centrifugation (14000 RPM, 10 min, 4°C) and total protein concentration was determined by Bradford assay.

Capture ELISA was done using a polyclonal rabbit anti-MMP11 antibody (Abcam, ab53143) coated onto 96 well plates (Nunc Maxisorp) at 1 μg/ml over night at 4°C. Wells were blocked with Tris-buffered solution, 0.05% Tween 20 (TBST) and 3% bovine serum albumin (blocking buffer), 100 μL per well for 2 hours at room temperature. The wells were incubated with patient plasma diluted 1:10 and 1:100 in TBST + 1% bovine serum albumin for 2 hours at room temperature. After washing three times in TBST, a mouse monoclonal anti-MMP11 antibody (Novus Biologicals, SL3-01) was added to the wells at a 1:100 dilution in blocking buffer and incubated for 2 hrs at room temperature. An HRP conjugated anti-mouse IgG (Abcam) diluted 1/1000 was used as detection agent following 3 washings in TBST. TMB developing reagent (Pierce) was added and the reaction stopped with 1 N HCl and the absorbance read at 450 nm. MMP11 levels were quantitated by comparison to a standard curve using a commercial MMP11 protein (Abcam, ab92861) at several dilutions. The assay specificity was optimized as described in Additional file 1: Figure S1. The sensitivity in different biological fluids such as cell supernatants or human plasma was determined as described in Additional file 2: Figure S2. SuperBlock blocking buffer (Pierce) and LI-COR buffer (LI-COR Biosciences) were also evaluated. The assay sensitivity was about 50 ng/ml.

### Detection of anti-MMP11 autoantibodies

Recombinant human MMP11 protein (Abcam) was coated at 100 ng/well onto 96 well plates (Nunc Maxisorp) in PBS over night at 4°C. Wells were blocked with PBS, 3% bovine serum albumin, 100 μL per well for 2 hours at room temperature. Plasma samples were added at 1:20 to 1:540 dilution in PBS, 1% bovine serum albumin, 100 μL per well and incubated over night at 4°C. The day after, an AP-conjugated anti-human IgG (Sigma) diluted 1:2000 in PBS, 1% bovine serum albumin and incubated at room temperature for 1 hr was used as detection agent. The Alkaline Phosphatase Yellow (pNPP) liquid substrate system (Sigma) was used and absorbance measured at 405 nm.

## Results and discussion

### MMP11 protein expression in invasive breast and prostate cancer

**Figure 1:**
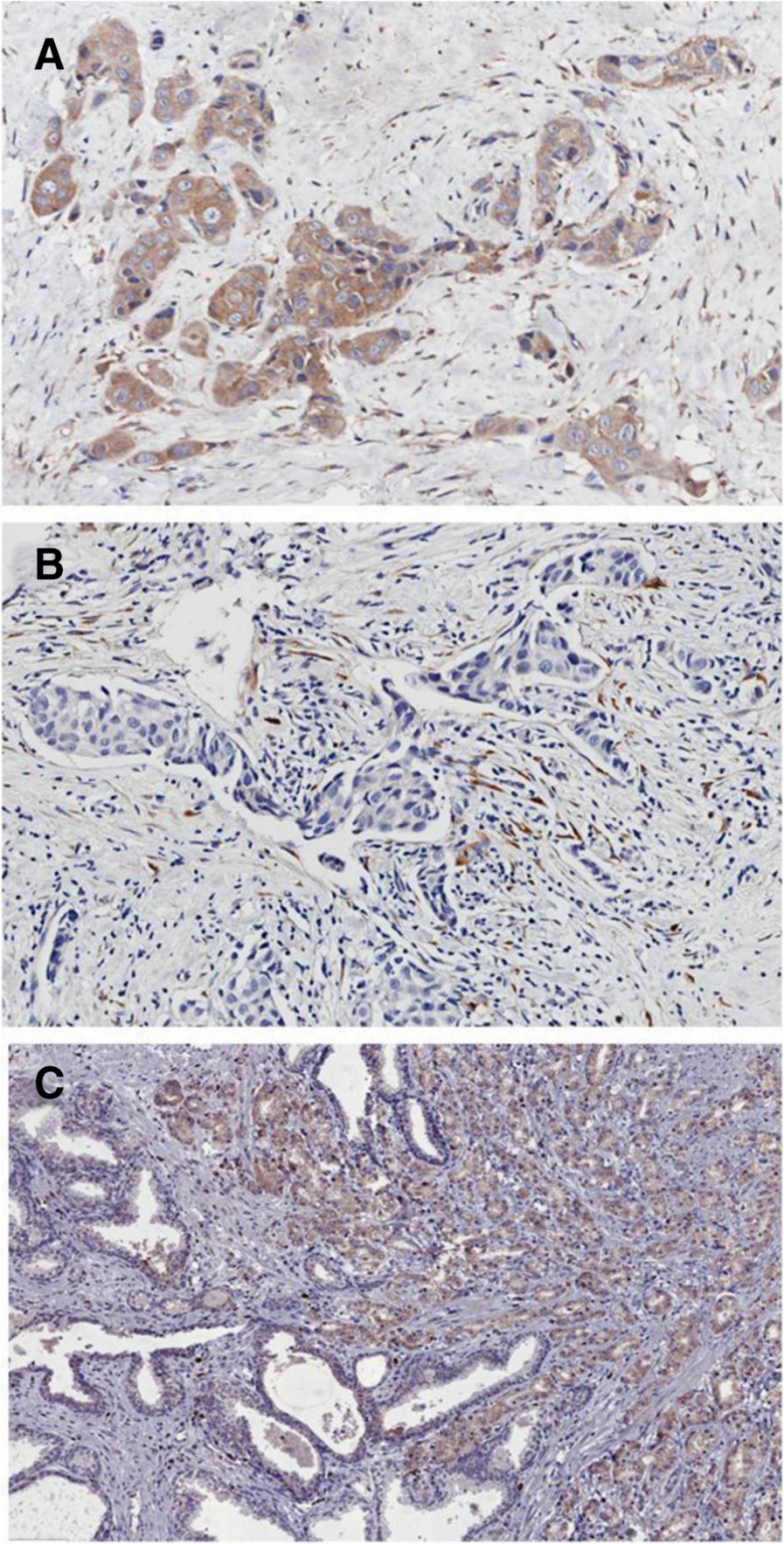
**Representative images of immunostaining with anti-MMP11 in breast (A, B) and in prostate (C) cancer (original magnification x200).** In **A** immunoreactivity for MMP11 was observed in the cytoplasm of the invasive ductal carcinoma cells and in the fibrous stroma. In **B** only peritumoral fibroblasts were positive. In **C** prostate cancer cells showed a strong cytoplasmic staining; intermingled normal glands were completely negative or faintly positive.

In order to evaluate MMP11 protein expression in invasive breast and prostate cancer, 11 HER2-positive invasive ductal carcinoma of the breast and 5 invasive prostate adenocarcinomas were immunostained for MMP11 (Figure 1). Positive cytoplasmic staining was observed in 3/11 breast carcinomas (Figure 1A). Of the remaining cases 3 showed immunoreactivity only in the peritumoral fibroblasts (Figure 1B), and 5 were completely negative. No significant staining was observed in normal mammary glands. Prostate cancers showed a strong cytoplasmic staining in 3/5 cases (Figure 1C). Normal prostate glands were completely negative or occasionally weakly positive.

### Cancer patients characteristics

We collected 13 plasma samples from breast cancer patients and 12 from prostate cancer patients. In addition, we used 7 samples from healthy donors as negative controls. All breast cancer patients had HER-2-positive histologically confirmed primary invasive breast adenocarcinoma with no evidence of residual, locally recurrent, or metastatic disease after completion of surgery and chemotherapy (neoadjuvant or adjuvant), an ECOG performance status of 0 or 1, and were under Trastuzumab treatment.

Prostate cancer patients had documented non metastatic castration-resistant prostate cancer determined by increasing serum PSA despite castrate levels of testosterone (<50 ng/dL), with no radiographic evidence of metastatic disease. Mean Gleason score 7 (range: 3–9).

### Detection of circulating MMP11 protein

**Figure 2:**
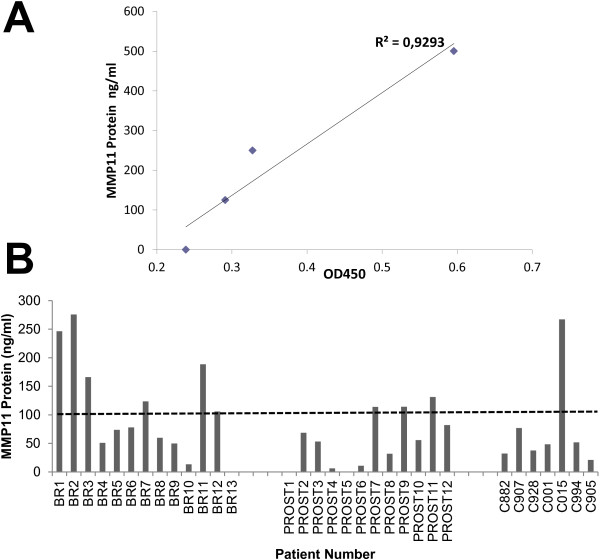
**MMP11 detection in plasma samples. A)** standard curve of the ELISA assay. MMP11 recombinant protein was diluted in 1:10 dilution of a normal control plasma. **B)** MMP11 was measured by ELISA in breast (BR) and prostate (PROST) cancer plasma samples as described in Methods. Control (C) samples were obtained from healthy donors. The assay was run in duplicate and repeated twice, with a inter-variability of 7.6%. A threshold at 100 ng/ml was determined (see text) to identify positive and negative plasma samples.

As previously shown, MMP11 expression in breast and prostate cancer was confirmed by IHC in a high percentage of cases with similar characteristics (Figure 1). Among the metalloproteases, MMP11 is the only enzyme secreted in an active form [15]. To date, an assay to specifically detect and quantify MMP11 catalytic activity in biological samples has not yet been established. To see whether the protein could be detected in the blood, we have set up an ELISA assay (see Methods) by means of two commercial antibodies and adapted it to different biologic fluids, such as cell extracts, supernatants and blood plasma. To establish the assay with a protein endowed of similar features and post-translational modifications found in patients, the assay was optimized using cell extracts derived from HeLa transfected with a human MMP11 expression vector [10]. Results are shown in Additional file 1: Figure S1 and Additional file 2: Figure S2 and indicate that plasma matrix does not affect the overall assay performance and sensitivity. Based on this observation, MMP11 was evaluated in 13 breast and 12 prostate cancer plasma samples, respectively, compared to a healthy donor cohort. To define a standardized threshold of the ELISA assay, a signal mean value plus 3 times the standard deviation was calculated from 6 healthy donors, corresponding to 102.7 ng/ml (44.7 + 3*19.34 ng/ml). In this setting, circulating MMP11 was detected at different levels in 5 out of 13 breast cancer patients as well as in 3 out of 12 prostate cancer patients. A lower expression level was measured in healthy donors with one single exception (C015, Figure 2).

### Antibodies against MMP11 and clinical outcome

**Figure 3:**
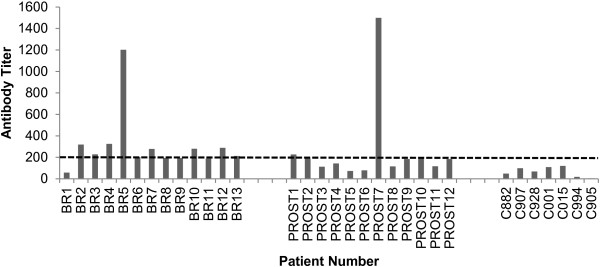
**Anti-MMP11 detection in plasma samples.** MMP11 antibodies were measured by ELISA. Anti-MMP11 titers were calculated as the reciprocal limiting dilution of plasma producing an absorbance at least 3-fold greater than the absorbance of control samples average at the same dilution. The assay was run in duplicate and repeated twice. A threshold at 1:200 was arbitrarily set to identify seropositive patients. BR, breast; PROST, prostate; C, controls.

To assess whether MMP11 could spontaneously be recognized as an antigen by the immune system, MMP11 recombinant protein was immobilized and used to titrate specific IgG antibodies in this cohorts of patients. An antibody response was observed in 7/13 breast cancer patients and in 2/12 prostate cancer patients (Figure 3). No antibodies (0/6) were measured in healthy donors. No association between MMP11 expression in the blood and the presence of specific antibodies was found.

## Conclusions

TAAs are important targets for immunization strategies and for the development of therapeutic antibodies. Targeting the tumor stroma as a cancer therapeutic approach has been established in several experimental and clinical studies. Matrix metalloproteinases (MMPs) have the desired properties as they are important components of tumor stroma and are present in almost all human cancers compared with normal tissue [6,7].

In this study, we have confirmed the expression of MMP11 in breast and prostate cancer (Figure 1) and for the first time we have found its expression in bloodstream and spontaneous autoantibodies in breast and prostate cancer patients (Figures 2 and 3). The prognostic significance of MMP11 expression for breast cancer was recently confirmed by Cheng *et al.*[13]. Overexpression of MMP-11 correlates with patients having poorly differentiated tumors, lymph node metastasis and lacking progesterone receptor. Temporally increased MMP-11 expression can be considered as an early event, occurring prior to lymph node metastasis during breast cancer progression. Similarly, MMP11 expression in prostate cancer patients was significantly correlated with poor differentiation in Gleason grading, pathologic tumor stage4 (pT4), and positive-bone metastasis (p < 0.05), but not age and prostatic-specific antigen (PSA) level. Patients with high levels of MMP-11 expression demonstrated significantly shorter survival (p < 0.001) when compared to those with low levels [14]. Therefore, high levels of MMP11 may potentially be used for prediction of a poor prognosis.

Our data show that MMP11 is indeed overexpressed in a subset of breast and prostate cancer patients. In our breast cancer specimens we were able to detect the expression either by the cancerous cells (Figure 1A) or by the peritumoral fibroblasts (Figure 1B). We also found a strong signal in 3/5 prostate cancer samples (Figure 1C). The presence of autoantibodies is in line with this finding. We are currently developing an assay to specifically detect and quantify MMP11 catalytic activity on a synthetic substrate peptide. Such assay will be instrumental to assess whether spontaneous and induced antibodies against MMP11 could have a biological role at inhibiting its enzymatic activity. Moreover, it will be of interest to find association among the circulating protein, the antibody titer and patients survival. We are currently following up these patients, accumulating new data and analyzing the IgG subtype to find potential associations. A limitation of our study is the restricted data set and the lack of match between MMP11 tumor expression and plasma samples within the same patients population. For this reason, our finding needs to be followed by a confirmatory study with a larger cohort of patients. Moreover, the observation that one healthy donor (C015, Figure 2) showed a high level of circulating MMP11 suggest that the protein may be involved in other biologic processes and indicate that larger patients cohorts and relative controls should be analyzed. In addition, we are generating preclinical data in mouse models that suggest that cell mediated immune response may play an important role as effective arm of the immune response as a consequence of an anti-MMP11 vaccination. A novel T-cell epitope derived from human MMP11 (hMMP_237_) was identified in our lab by vaccination of HLA-A2.1 (HHD) transgenic mice and was shown to be immunogenic by *in vitro* priming with human PBMCs. Moreover, activated CTLs secrete granzyme B, a key mediator of target cell death via the granule-mediated pathway [10]. Thus, the immune response against hMMP_237_ represents a potential biomarker for induced and spontaneous immune response. We are currently analyzing by tetramer staining and *in vitro* priming the T cell responses in PBMCs from patients affected by different tumor types, including breast and prostate cancer. It will be of interest to correlate IHC, circulating protein, antibodies and T cell responses with clinical behavior and survival outcome.

In conclusion, our study, albeit preliminary, further suggest that MMP11 may act as a *bona fide* TAA and be a suitable target for cancer immunotherapy.

## Abbreviations

MMP11: Matrix metalloproteinase 11; CEA: Carcinoembryonic antigen.

## Competing interests

The author declares that they have no competing interests.

## Authors’ contribution

GR performed protein quantitation assay and its setting up; MC performed the antibody titration assay as well as data analysis; CDV, ADN and LR provided control samples and performed IHC studies; RM and GC contributed to data analysis and draft of sections of the manuscript; LA conceived and supervised the study, and revised the manuscript. All authors read and approved the final version of the manuscript.

## Supplementary Material

Additional file 1: Figure S1 Set up and Optimization of the ELISA assay for detection of MMP11 protein. HeLa cells (MMP11 negative) were plated onto 6 cm dishes and transfected with an expression vector for MMP11 [10]. Two days later, cell lysates were prepared and incubated at the indicated amount O/N in a 96 well plate previously coated with a polyclonal rabbit anti-MMP11 antibody. After washing, plates were incubated with a monoclonal mouse anti-MMP11 antibody and the detection was executed with an anti-mouse IgG-HRP. Blocking and incubation were performed in A) TBST + 1% BSA; B) TBST + 5% milk; C) SuperBlock buffer; D) LICOR Blocking Buffer (BB), 0.1% Tween 20. E) a direct comparison of the influence of the buffer on specific (HeLa transfected) vs non-specific (HeLa mock treated) signal. The best conditions were obtained with TBST + 1% BSA. HeLa NT indicates mock transfected cells; HeLa-hMMP11 indicates cells transfected with the expression vector. Click here for file

Additional file 2: Figure S2 Sensitivity of the assay in different biologic fluids. The assay was run with the conditions defined in Additional file 1: Figure S1. MMP11 recombinant protein was diluted in TBST + 1% BSA, cell culture medium (DMEM, 10% FCS) or TBST + human plasma diluted 1:10. The signal at higher concentrations was reduced of about 30% in the presence of plasma, but the sensitivity was similar in the three conditions. The assay was run in triplicates and repeated twice with similar results. Click here for file
