# Supplementary material for: Circulating MMP11 and specific antibody immune response in breast and prostate cancer patients
Source: J Transl Med. 2014 Feb 24;12:54. doi: 10.1186/1479-5876-12-54 (PMC3936832; doi:10.1186/1479-5876-12-54)
Supplement: Additional file 1: Figure S1 — Set up and Optimization of the ELISA assay for detection of MMP11 protein. HeLa cells (MMP11 negative) were plated onto 6 cm dishes and transfected with an expression vector for MMP11 [10]. Two days later, cell lysates were prepared and incubated at the indicated amount O/N in a 96 well plate previously coated with a polyclonal rabbit anti-MMP11 antibody. After washing, plates were incubated with a monoclonal mouse anti-MMP11 antibody and the detection was executed with an anti-mouse IgG-HRP. Blocking and incubation were performed in A) TBST + 1% BSA; B) TBST + 5% milk; C) SuperBlock buffer; D) LICOR Blocking Buffer (BB), 0.1% Tween 20. E) a direct comparison of the influence of the buffer on specific (HeLa transfected) vs non-specific (HeLa mock treated) signal. The best conditions were obtained with TBST + 1% BSA. HeLa NT indicates mock transfected cells; HeLa-hMMP11 indicates cells transfected with the expression vector. [file 1479-5876-12-54-S1.pptx]

## Slide 1
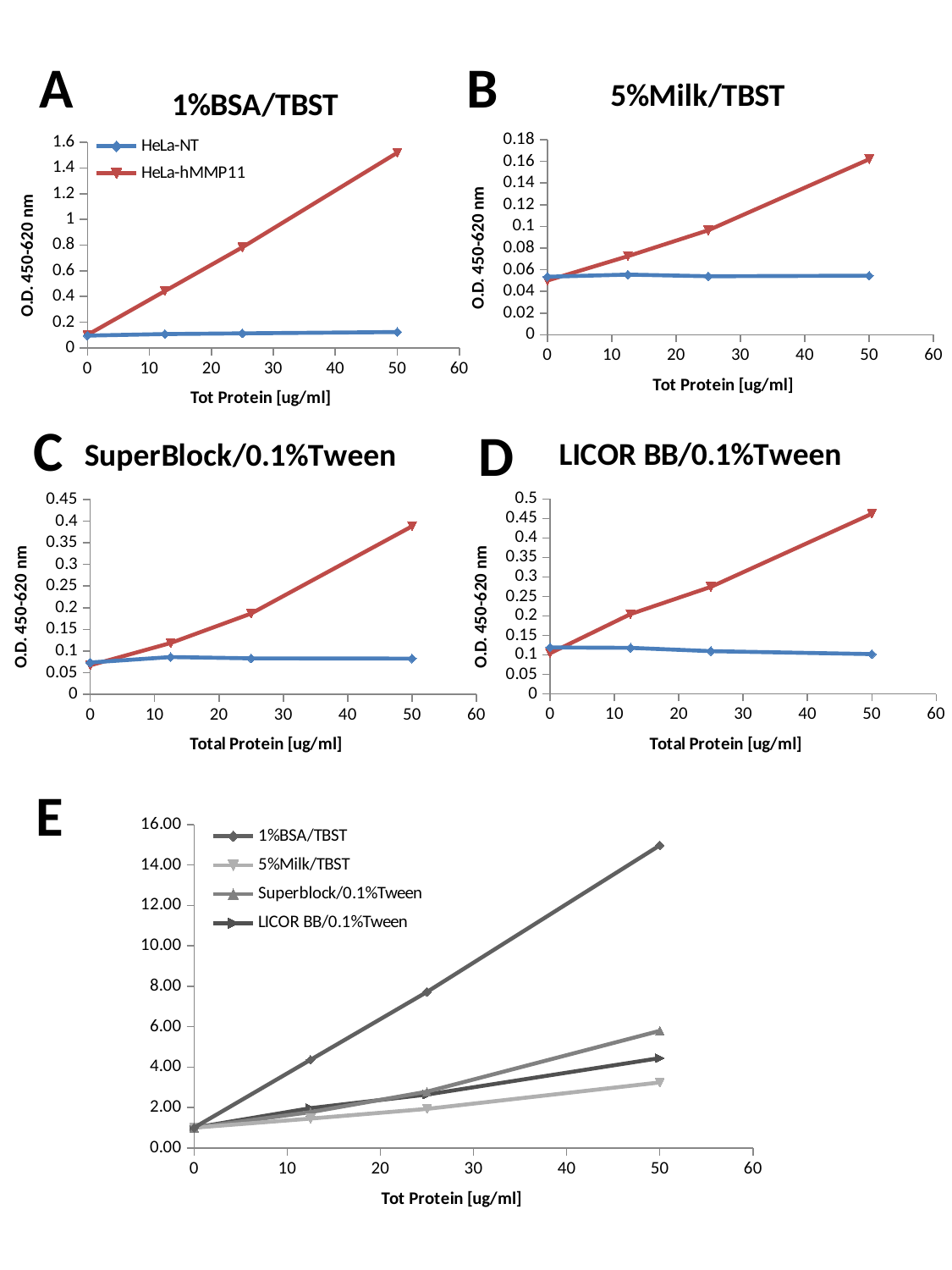

A
B
### Chart: 5%Milk/TBST
| Category | HeLa-NT | HeLa-hMMP11 |
|---|---|---|
### Chart: 1%BSA/TBST
| Category | HeLa-NT | HeLa-hMMP11 |
|---|---|---|C
D
### Chart: LICOR BB/0.1%Tween
| Category | HeLa-NT | HeLa-hMMP11 |
|---|---|---|
### Chart: SuperBlock/0.1%Tween
| Category | HeLa-NT | HeLa-hMMP11 |
|---|---|---|E
### Chart
| Category | 1%BSA/TBST | 5%Milk/TBST | Superblock/0.1%Tween | LICOR BB/0.1%Tween |
|---|---|---|---|---|
