# Supplementary figures and images for: Circulating MMP11 and specific antibody immune response in breast and prostate cancer patients
Source: J Transl Med. 2014 Feb 24;12:54. doi: 10.1186/1479-5876-12-54 (PMC3936832; doi:10.1186/1479-5876-12-54)

## Slide 1
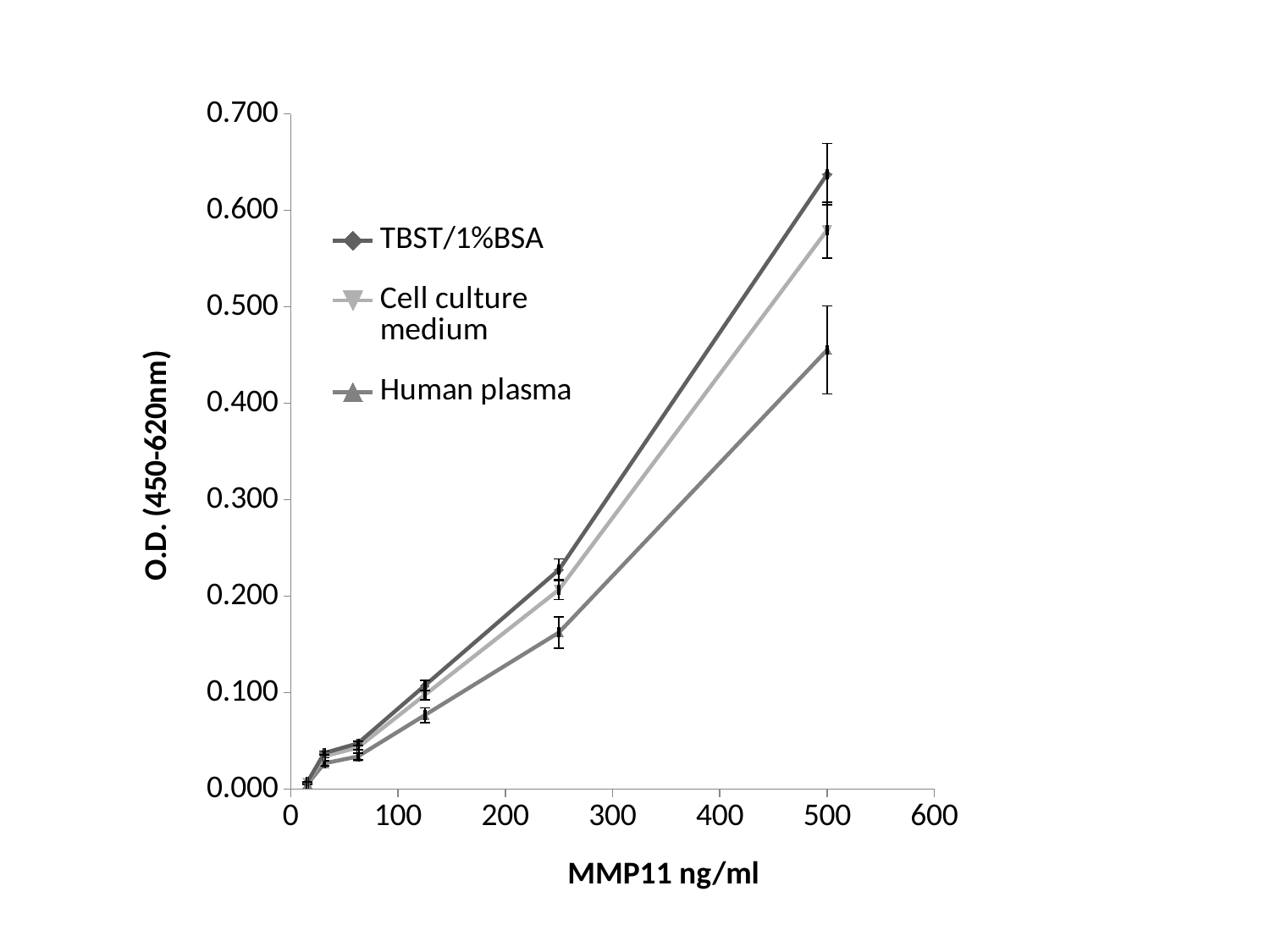

### Chart
| Category | TBST/1%BSA | Cell culture medium | Human plasma |
|---|---|---|---|MMP11 ng/ml

Supplement: Additional file 2: Figure S2 — Sensitivity of the assay in different biologic fluids. The assay was run with the conditions defined in Additional file 1: Figure S1. MMP11 recombinant protein was diluted in TBST + 1% BSA, cell culture medium (DMEM, 10% FCS) or TBST + human plasma diluted 1:10. The signal at higher concentrations was reduced of about 30% in the presence of plasma, but the sensitivity was similar in the three conditions. The assay was run in triplicates and repeated twice with similar results. [file 1479-5876-12-54-S2.pptx]
